# Shared ancestral susceptibility to colorectal cancer and other nutrition related diseases

**DOI:** 10.1186/1471-2350-13-94

**Published:** 2012-10-05

**Authors:** Stefanie Huhn, Melanie Bevier, Anja Rudolph, Barbara Pardini, Alessio Naccarati, Michael Hoffmeister, Ludmila Vodickova, Jan Novotny, Hermann Brenner, Jenny Chang-Claude, Kari Hemminki, Pavel Vodicka, Asta Försti

**Affiliations:** 1 Department of Molecular Genetic Epidemiology, German Cancer Research Center DKFZ, Heidelberg 69120, Germany; 2 Division of Cancer Epidemiology, German Cancer Research Center DKFZ, Heidelberg 69120, Germany; 3 Institute of Experimental Medicine, Academy of Sciences of the Czech Republic, Prague, 14200, Czech Republic; 4 Division of Clinical Epidemiology and Aging Research, German Cancer Research Center DKFZ, Heidelberg, 69120, Germany; 5 Institute of Biology and Medical Genetics, 1st Faculty of Medicine, Charles University, Prague 2, 12000, Czech Republic; 6 Department of Oncology, General Teaching Hospital, Prague 2, 12808, Czech Republic; 7 Center of Primary Health Care Research, Clinical Research Center, Lund University, Malmö, SE-20502, Sweden; 8 PMV Forschungsgruppe, University of Cologne, Cologne, 50931, Germany

**Keywords:** Colorectal cancer, Nutrition, Complex diseases

## Abstract

**Background:**

The majority of non-syndromic colorectal cancers (CRCs) can be described as a complex disease. A two-stage case–control study on CRC susceptibility was conducted to assess the influence of the ancestral alleles in the polymorphisms previously associated with nutrition-related complex diseases.

**Methods:**

In stage I, 28 single nucleotide polymorphisms (SNPs) were genotyped in a hospital-based Czech population (1025 CRC cases, 787 controls) using an allele-specific PCR-based genotyping system (KASPar®). In stage II, replication was carried out for the five SNPs with the lowest p values. The replication set consisted of 1798 CRC cases and 1810 controls from a population-based German study (DACHS). Odds ratios (ORs) and 95% confidence intervals (CIs) for associations between genotypes and CRC risk were estimated using logistic regression. To identify signatures of selection, Fay-Wu’s H and Integrated Haplotype Score (iHS) were estimated.

**Results:**

In the Czech population, carriers of the ancestral alleles of *AGT* rs699 and *CYP3A7* rs10211 showed an increased risk of CRC (OR 1.26 and 1.38, respectively; two-sided p≤0.05), whereas carriers of the ancestral allele of *ENPP1* rs1044498 had a decreased risk (OR 0.79; p≤0.05). For rs1044498, the strongest association was detected in the Czech male subpopulation (OR 0.61; p=0.0015). The associations were not replicated in the German population. Signatures of selection were found for all three analyzed genes.

**Conclusions:**

Our study showed evidence of association for the ancestral alleles of polymorphisms in AGT and CYP3A7 and for the derived allele of a polymorphism in ENPP1 with an increased risk of CRC in Czechs, but not in Germans. The ancestral alleles of these SNPs have previously been associated with nutrition-related diseases hypertension (AGT and CYP3A7) and insulin resistance (ENPP1). Future studies may shed light on the complex genetic and environmental interactions between different types of nutrition-related diseases.

## Background

Colorectal cancer (CRC; OMIM ID: 114500) is among the most common cancers in industrialized countries and one of the leading causes of cancer-related mortality [1]. The incidence rates vary among different groups and populations depending on sex, age and country, with higher rates among males than females and increasing with age [2]. The differences in the incidence rates across the globe are mainly attributed to differences in diet and other environmental factors.

In both sporadic and familial CRC, genes and environment together contribute to the risk of CRC. The majority of non-syndromic CRCs can be characterized as a complex disease [3]. The major factors that modify CRC risk are obesity, diabetes, red meat consumption, physical inactivity, alcohol consumption, chronic inflammation, and cigarette smoking [4-6]. Additionally, the intake of vitamin D, calcium, fruit and vegetables may potentially influence the risk of CRC [5]. Gene-environment interactions also underlie other complex diseases, such as obesity (OMIM ID: 601665) and diabetes mellitus type II (T2D; OMIM ID: 125853).

A specific feature of CRC and other complex diseases is that they are mainly diseases of humans living in industrialized societies, in environments with almost unlimited food supply and low energy expenditure [5,7]. Nutrition is one of the most important environmental traits influencing the fitness of an individual. In the past centuries, the genetic constitution of an individual was supposed to optimize food utilization in order to protect against malnutrition. In modern societies, the ancestral genetic constitution might not be beneficial anymore, because it does not protect against the relatively new condition of overnutrition. Therefore, variants that protect against overnutrition and related health issues are supposed to be rare [7]. Nevertheless, genetic variants that promote a carbohydrate-based nutrition as well as genetic variants that show ancestral susceptibility to a nutrition-related disease have already been described (inter alia [8-10]). Signatures of such processes can be detected in the human genome using genome-wide approaches that evaluate differences in the world-wide allele frequencies and haplotype distribution (inter alia [11-15]).

Based on the interplay between genetic and environmental risk factors in nutrition-related complex diseases we posed the following hypothesis: “Polymorphisms with ancestral alleles associating with a nutrition-related disease and showing signatures of recent selection may be associated with CRC risk.” We outline here the methods used for selecting such candidate genes and show the results when the selected variants were tested for CRC risk in two large case–control studies.

## Methods

### Candidate SNP selection for the case–control study

The study focused on SNPs, for which ancestral alleles have previously been associated with nutrition-related complex diseases other than CRC, such as obesity, T2D and metabolic syndrome. Information about such SNPs was collected from 30 published reports by browsing the PubMed database (http://www.ncbi.nlm.nih.gov/sites/entrez?db=pubmed) [16] for the keywords “diabetes”, “obesity”, “metabolic syndrome” (OMIM ID: 605552) and “hypertension” (OMIM ID: 145500) up to 06/2009. Most of the articles were based on genome-wide association studies or were meta-analyses. A complete list of the publications can be found at the reference list of the Additional file1.

From these 30 reports, associations with the risk of the diseases and with the related quantitative traits were retrieved. The quantitative traits for diabetes were fasting glucose level and insulin resistance. For obesity, the traits were body mass index (BMI) and waist to hip ratio. The quantitative traits for hypertension and the metabolic syndrome were high-density lipoprotein (HDL) level, low-density lipoprotein (LDL) level, triglycerides level, salt sensitivity, blood pressure and insulin resistance. A complete list of the reported associations can be found in the Additional file 1.

**Figure 1:**
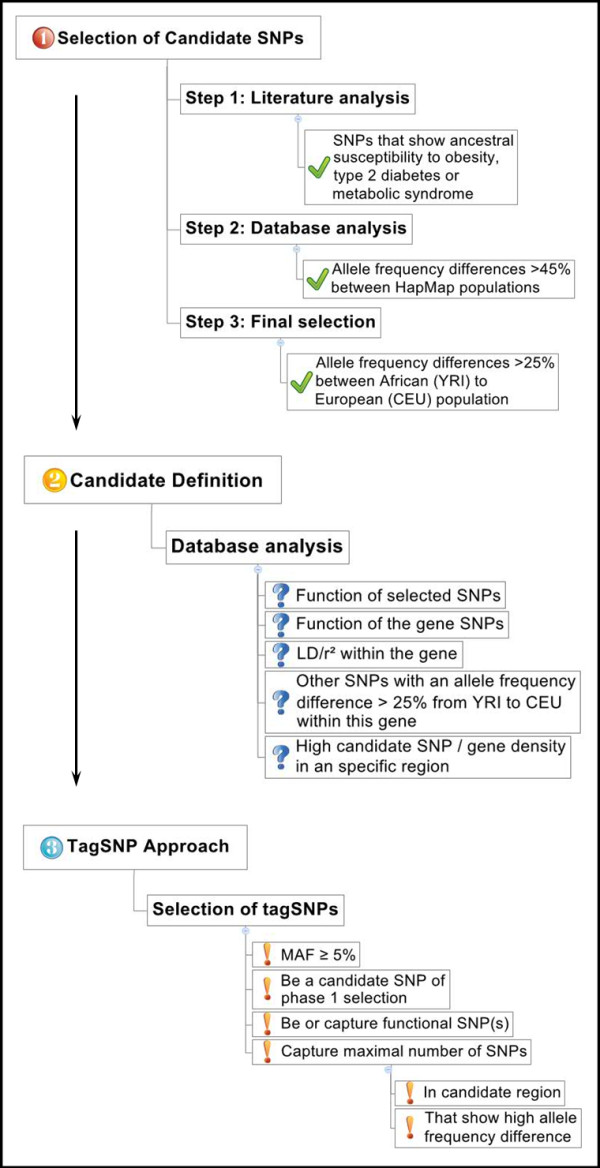
Workflow for the selection of candidate SNPs for the ancestral susceptibility project.

The candidate SNP selection for the association study took place in three major phases (Figure 1):

(1) “Selection of Candidate SNPs”: All published SNPs were evaluated for the nature of the risk allele – either ancestral (A) or derived (D) - and the allele frequency differences between African, European and Asian populations (YRI: Sub-Saharan African population, Yoruba in Ibadan, Nigeria; CEU: Caucasian population, Utah residents with Northern and Western European ancestry from the CEPH collection; HCB: East Asian population, Han Chinese in Beijing, China; JPT: East Asian population, Japanese in Tokyo, Japan). An allele was considered a “risk allele” when it was associated with a significantly increased risk of a key-disease (OR>1; statistical significance based on the criteria of the original publication), or when it was associated with a significant increase of quantitative values in the original publication. The nature of the risk allele was determined by using the NCBI database (http://www.ncbi.nlm.nih.gov) [16].

The reported ancestral susceptibility SNPs that showed an absolute allele frequency difference of >45% between the African and any non-African population were chosen for further investigation. The threshold value of 45% was set to detect variants with a “major-to-minor” allele change between populations, thus indicating a possible influence of selective pressure. A second, lower threshold (25%) was set for the difference between the YRI and the CEU population to acknowledge the more recent separation of the European than the East Asian population from the African population.

(2) “Candidate Gene Definition”: The SNPs that passed the first selection criteria were evaluated for their location in the genome, possible functional effects, linkage disequilibrium (LD) with other polymorphisms within the gene region and the number of candidate SNPs in the gene region.

(3) “Tagging SNP Approach”: In addition to the evaluation of the reported ancestral susceptibility SNPs, a tagging SNP approach was carried out for each candidate gene or gene region using the genotyping data of the CEU population and HaploView^©^ software [17]. Next to a minor allele frequency (MAF) of ≥5%, a tagging SNP had to feature the following parameters:

– be or capture a phase 1 SNP and/or

– be or capture a functional polymorphism and/or

– capture a maximal number of SNPs within a candidate gene or gene region with >25% allele frequency difference between the YRI and the CEU population

In the majority of cases, the reported ancestral susceptibility SNP itself was genotyped. When that was not possible (e.g. because the assay design failed due to the structure of the surrounding sequence) another SNP that was in LD with the reported SNP (r^2^>0.9) was selected for genotyping in order to indirectly gain information about the reported ancestral susceptibility SNP. For large, diverse genes/gene clusters, additional tagging SNPs were selected in order to gain more knowledge about the genes. These tagging SNPs should also fulfil the criterion of >25% allele frequency difference between the YRI and CEU population (Table 1).

**Table 1:** Information about genes and SNPs selected for the SNP association study

**Closest Candidate Gene**	**rs Number**^**a**^**(r**^**2**^**)**^**b**^	**Status (reported / genotyped)**	**Position**	**SNPs**^**c**^**(ancestral / derived)**	**Reported association**^**d**^	**Max. allele frequency difference (YRI vs. any)**	**Allele frequency difference YRI vs. CEU**	**Function or location**	**Ref.**
***AGT*** Angiotensin I	**rs699**	R/G	Chr1: 228.912.667	**C**/T	Hypertension, Preeclampsia	52,5%	52,5%	missense	[9,18]
	rs4762	R	Chr1: 228.912.850	**C**/T	Hypertension	3,3%	3,3%	missense	
	rs5051 (r^2^ 0.948 to rs699)	R	Chr1: 228.916.745	**A**/G	Hypertension	52,1%	52,1%	5′UTR	
***GALNT2*** Polypeptide N-acetylgalactosaminyl-transferase 2	**rs4846914**	R/G	Chr1: 228.362.564	**G**/A	HDL	58,0%	58,0%	intron	[19,20]
	**rs611229**	G	Chr1: 228.390.690	G/T	tagging SNP	39,4%	39,4%	intron	-
	rs2144300 (r^2^ 0.933 to rs4846914)	R	Chr1: 228.361.789	**C**/T	HDL, Triglycerides	57,0%	57,0%	intron	[20]
***IFIH1*** Interferon induced with helicase C domain 1	**rs1990760**	R/G	Chr2: 162.832.547	**C**/T	T2D	54,0%	54,0%	missense / benign	[21]
***SLC2A*** Solute carrier family 2, facilitated glucose transporter member 2	**rs5400**	R/G.	Chr3: 172.215.244	**T**/C	T2D	48,1%	35,0%	missense / damaging	[9]
	**rs6785233**	G	Chr3: 172.239.679	G/T	tagging SNP	50,8%	38,3%	-	-
	**rs8192675**	G	Chr3: 172.207.577	G/A	tagging SNP	76,7%	50,0%	-	-
***IGF2BP2*** Insulin-like growth factor 2 mRNA binding	**rs1470579**	R/G	Chr3: 187.012.024	**C**/A	T2D	60,0%	56,8%	intron	[22]
***ENPP1*** Ectonucleotide pyrophosphatase	**rs1044498**	R/G	Chr6: 132.214.311	**C**/A	Insulin resistance, T2D (early onset), Myocardial infarction (early onset)	94,4%	87,3%	missense / benign	[9,23]
	**rs9493119**	G	Chr6: 132.253.111	G/A	tagging SNP	30,0%	20,8%	-	-
***CYP3A_ Locus*** Cytochrom P450	**rs776746**	R/G.	Chr7: 99.108.725	**A**/G	Hypertension; Salt sensitivity	79,2%	79,2%	CYP3A5_ intron	[9,18]
	**rs10211**	G	Chr7: 99.140.930	G/A	tagging SNP	67,0%	67,0%	3′UTR_CYP3A7	-
	**rs667660**	G	Chr7: 99.257.162	C/A	tagging SNP	74,3%	74,3%	upstream CYP3a3	-
***MTMR9*** Myotubularin related protein 9	**rs10091637**	G	Chr8: 11.181.724	C/T	tagging SNP	36,9%	29,2%	intron	-
	**rs11250127**	G	Chr8: 11.207.619	G/A	tagging SNP	66,4%	26,5%	intron	-
	rs2293855 (r^2^ 1.00 to rs11250127)	R	Chr8: 11.215.070	**G**/A	Obesity	64,2%	25,2%	intron	[24]
***ABCA1*** ATP-binding cassette transporter	**rs4149268**	R/G.	Chr9: 106.687.291	**T**/C	HDL	51,0%	46,0%	intron	[20]
	rs4149274	R	Chr9: 106.679.485	**A**/G	HDL	4,0%	2,0%	intron	[20]
	rs1883025	R	Chr9: 106.704.372	**T**/C	HDL	13,5%	10,0%	intron	[19]
***GAD2*** Glutamate decarboxylase 2	**rs2236418**	R/G.	Chr10: 26.545.752	**G**/A	Obesity	74,9%	74,9%	5′UTR	[9]
	**rs2839670**	G	Chr10: 26.544.346	A/C	tagging SNP	60,0%	60,0%	upstream	-
	**rs6482538**	G	Chr10: 26.549.870	G/A	tagging SNP	63,6%	63,6%	intron	-
	**rs7076544**	G	Chr10: 26.597.731	G/A	tagging SNP	68,0%	68,0%	intron	-
	**rs8190748**	G	Chr10: 26.609.761	G/A	tagging SNP	60,20%	60,20%	intron	-
**Chr 12q13 *ERBB3*** Avian erythroplastic leukemia viral oncogen homolog 3	**rs11171739**	R/G	Chr11: 54.757.142	**C**/T	T2D	56,0%	48,0%	ERBB3 ~4kb downstream	[21]
***KCTD10*** Potassium channel tetramerisation domain	**rs7298565**	G	Chr12: 108.421.917	A/G	tagging SNP	57,7%	24,1%	missense	-
	rs2338104 (r^2^ 0.98 to rs7298565)	R	Chr12: 108.379.801	**G**/C	HDL	45,0%	25,0%	KCTD10 intron; MYO1H ~9kb upstream	[19,20]
***CYP19A1 / GLDN*** Cytochrom P450 / Gliomedin	**rs2446405**	R/G	Chr15: 49.434.335	**A**/T	Insulin Resistance	43,0%	43,0%	GLDN intron	[25]
	**rs12592797**	G	Chr15: 49.426.928	A/C	tagging SNP	52,5%	52,5%	GLDN intron	-
	**rs2445761**	G	Chr15: 49.402.908	C/T	tagging SNP	40,9%	40,9%	CYP19A1 intron	-
***BBS4*** Bardet-Biedl syndrome-4	**rs7178130**	R/G	Chr15: 70.765.505	**G**/A	Bardet-Biedl Syndrome; Obesity	50,0%	50,0%	upstream 5^′^	[26]
***C18orf8 / NCP1*** Chromosome 18 open reading frame 8 / Niemann-Pick disease, type C1	**rs891386**	R/G	Chr18: 19.357.969	A/C	tagging SNP	42,5%	42,5%	CRC associated Protein	[27]
	rs1805081 (r^2^ 0.84 to rs891386)	R	Chr18: 19.394.680	**A**/G	BMI	46,7%	46,7%	missense	

R reported SNP; G genotyped SNP; “R/G” reported and genotyped SNP. Max. maximal; BMI body mass index, T2D type 2 diabetes, LDL low-density lipoprotein, HDL high-density lipoprotein, Ref. reference ^a^ genotyped SNPs are indicated in bold; ^b^ pair-wise linkage disequilibrium between the SNPs, if reported SNP was not genotyped directly; ^c^ reported risk allele is indicated in bold-underlined; ^d^ Next to the keywords that were used to collect and select the candidate SNPs from the literature, the table also includes all reported associations with related quantitative traits.

Allele frequencies were obtained from the NCBI database for the submitter population IDs: HapMap -CEU, -YRI, -HCB and -JPT (NCBI dbSNP Build 130; http://www.ncbi.nlm.nih.gov) [16]. LD/r^2^ was obtained from HapMap using the HapMap3 - and HapMart Genome Browser (Release #2, Phase 3; http://hapmap.ncbi.nlm.nih.gov) [28,29] and HaploView© software (Version 3, Release 2, Analyse Panel CEU) [17] with the implemented Tagger tagSNP selection algorithm [30]. The data about possible functionality originate from the NCBI database (http://www.ncbi.nlm.nih.gov) [16] and the PolyPhen (http://genetics.bwh.harvard.edu/pph/) [inter alia [31].

### Study populations

First, a hospital-based study population from the Czech Republic was analyzed. Between 09/2004 and 05/2009, 1025 CRC cases were recruited by nine oncological departments in the Czech Republic [32]. The sampled patients showed positive colonoscopic results for malignancy, histologically confirmed as colon or rectal carcinomas. The patients who met the Amsterdam criteria I or II for hereditary nonpolyposis colorectal cancer (OMIM ID: 120435) were excluded from the study. During the same time period, 787 healthy controls were recruited by five gastroenterological departments of the Czech Republic [32]. They were individuals undergoing colonoscopy for various gastrointestinal complaints, such as macroscopic bleeding, positive faecal occult blood test or abdominal pain of unknown origin. Only individuals showing negative colonoscopic results for malignancies, colorectal adenomas, benign polyps or inflammatory bowel disease were eligible for the study. Beside general information about sex and age, information about BMI (OMIM ID: 606641) and diabetes status was available for most of the individuals (Table 2).

**Table 2:** Characteristics of the Czech and the DACHS study population, at the time of diagnosis for cases and at the time of sampling for controls

**Characteristics**	**Czech Cases**	**Czech Controls**	**p value**	**DACHS Cases**	**DACHS Controls**	**p value**
total	1025	787		1798	1810	
male [n (%)]	594 [58%]	453 [58%]	0.35^c^	1052 [59%]	1078 [60%]	0.52^c^
female [n (%)]	399 [39%]	333 [42%]		746 [41%]	732 [40%]	
missing sex information [n (%)]	32 [3%]	1 [0.1%]		0		
median age [range]	63 [26–86]	55 [24–91]	<0.0001^b^	69 [33–94]	70 [34–98]	0.21^b^
missing age information [n (%)]	32 [3%]	1 [0.1%]		0		
median BMI^a^ [range]	27 [13–53]	26 [17-44]	0.49^b^	27 [17–50]	26 [16–46]	<0.0001^b^
missing BMI^a^ information [n (%)]	312 [30%]	294 [37%]		28 [2%]	10 [0.6%]	
diabetes [n (%)]	142 [14%]	62 [8%]	0.002^c^	326 [18%]	247 [14%]	0.0002^c^
diabetes no [n (%)]	598 [58%]	434 [55%]		1456 [82%]	1558 [86%]	
missing diabetes information [n (%)]	285 [28%]	291 [37%]		16 [0.9%]	5 [0.3%]	

BMI, body mass index; ^a^ for case samples BMI five years before diagnosis; ^b^ Wilcoxon two sample test; ^c^ chi-square test.

The SNPs that showed nominally significant associations in the Czech population were additionally analysed in a German population-based case–control study. The DACHS (Darmkrebs: Chancen der Verhütung durch Screening) study contributed 1798 cases and 1810 matched controls recruited from 01/2003 to 12/2007 in South-West Germany [33,34]. The patients included in the study had a first diagnosis of invasive CRC. As controls, individuals were randomly selected from lists of residents provided by the population registries. In the detailed standardized questionnaires, information about BMI at least five years before sampling and diabetes status was available in addition to general information about sex and age [34]. Table 2 outlines the characteristics of the Czech and the DACHS population relevant for the study.

### Ethical standards

The study was approved by the Ethics Committees of the Institute of Experimental Medicine, Academy of Sciences of the Czech Republic, Prague (Czech Republic); Institute for Clinical and Experimental Medicine and Faculty, Thomayer Hospital, Prague (Czech Republic); Medical Faculty of the University of Heidelberg (Germany) and the State Medical Boards of Baden-Württemberg and Rheinland-Pfalz (Germany). Written informed consent was obtained from all study participants.

### Genotyping

Genotyping was performed using a competitive allele-specific PCR genotyping system (KASPar®; KBiosciences, UK). PCR reactions were carried out in a 384-plate format using 3ng DNA per reaction in a 4μl reaction volume according to the optimal PCR protocol suggested by KBiosciences. The genotype detection was performed using an ABI PRISM 7900-HT Sequence Detection System with SDS 2.2 software (Applied Biosystems). For internal quality control, 7% of the Czech samples and 5% of the DACHS samples were randomly selected and included as duplicates. The concordance rate between the original and the duplicate samples was ≥ 99%. The average call rate was 98.2% in the Czech population and 97.1% in the DACHS population.

### Statistical analysis

The observed genotype frequencies in the controls were tested for Hardy-Weinberg equilibrium (HWE) using χ^2^ tests [35]. Odds ratios (ORs) and 95% confidence intervals (CIs) for associations between genotypes and CRC risk were estimated by logistic regression (PROC LOGISTIC, SAS Version 9.2; SAS Institute, Cary, NC) [36]. The estimated effects for all SNPs refer to the ancestral allele (A). Due to the low allele frequency shown by the majority of the tested polymorphisms the dominant model was applied in all estimations. The ORs were adjusted for age and sex. Additionally, a pooled analysis of the two studies was conducted. The ORs for the pooled analysis were adjusted for age, sex and study population.

Gene-gene interaction was studied for pair-wise interaction using logistic regression. To acknowledge the fact that men are in higher risk for CRC than women and that BMI is one of the most important risk factors for non-syndromic CRC, an analysis stratified by sex and BMI was performed. The threshold value for BMI was chosen according to the median BMI in the respective study population. To assess effect modification by sex and BMI, multiplicative interaction terms were utilized in multivariate regression models.

P values ≤ 0.05 were considered statistically significant. Bonferroni correction was not applied because it would have been overly conservative since the SNP selection was hypothesis-driven and all selected SNPs have previously been associated with a phenotype predisposing to CRC. Instead, a replication study using the German sample population was conducted to validate the initial results in the Czech population.

### Signatures of selection

Next to allele frequency differences between the African and the non-African populations, the study aimed to detect additional signatures of selection in those genes that were associated with CRC in the case–control study. Highly variable allele frequencies in different populations might be attributable to processes such as genetic drift, bottleneck events or founder effects that occur during the separation from the ancestral population. In order to encounter this problem, methods, which are less susceptible to demographic influences, were applied to investigate signatures of selective pressure. Instead of the traditional F_ST_ value and Tajiman D test, Fay-Wu’s H and the Standardized Integrated Haplotype Score (|iHS|) were estimated using the Haplotter web application that was developed on genome-wide HapMap data (http://haplotter.uchicago.edu/) [13,15]. The Fay-Wu’s H algorithm detects unusual excess of high frequency derived alleles in a gene region. Strong negative Fay-Wu’s H values are considered as signatures for a selective sweep [12,15]. The iHS measures the length of haplotypes around a given SNP in comparison to the whole genome. Values < −1.5 and > 1.5 (|1.5|) give conclusive evidence for natural selection while values < −2 or > 2 (|2.0|) give evidence for a powerful selection signal [15,37]. Values were estimated for the YRI, the CEU and the East Asian (ANS) population.

## Results

### Candidate SNP selection

With the keywords “diabetes”, “obesity”, “metabolic syndrome” and “hypertension”, we found in PubMed 30 publications, which reported 246 polymorphisms to be associated with the key-disease or with a related quantitative trait. Supplementary material (Additional file 1) provides information about all the 246 polymorphisms with the corresponding chromosomal position, allele status and frequency, reported associations with the diseases or the traits, information about the functionality of the SNPs and a complete reference list.

Carriers of the ancestral (A) allele of 130 SNPs had an increased risk of a key-disease (52.8%), whereas 106 SNPs were associated with an increased risk due to the derived (D) allele. The majority of the SNPs were located in introns (all 43.1%; A 48.5%; D 40.6%) or intergenic regions (all 38.6%; A 34.6%; D 47.2%). Less than 15% of all SNPs (A 17%; D 13%) were nonsense, missense, cds-synonymous or UTR SNPs.

The frequency differences estimated for the ancestral susceptibility alleles among the four worldwide populations were highly variable (YRI vs. any: mean 34.8; range 0.0-94.4; YRI vs. CEU: mean 20.7; range 0.0-87.3). Twenty-nine SNPs fulfilled the selection criteria of allele frequency difference >45% between the African and the non-African populations and >25% between the YRI and the CEU populations. These SNPs with their corresponding genes were considered as candidates for the CRC case–control study. After considering the location and the function of the SNPs and the LD characteristics of the gene regions, 28 SNPs in 15 genes were selected for the case–control study (Table 1).

### Association study

The genotype distribution of 27 of the 28 SNPs genotyped in the Czech control population was according to HWE. For *ERBB3* rs11171739, the genotype distribution deviated from HWE (p <0.0001) and the SNP was not considered in the further analyses. Except for *CYP3A5* rs776746, which was monomorphic in the Czech cohort, none of the observed allele frequencies differed significantly from the allele frequencies given in the NCBI database (CEU population).

Three SNPs in three genes showed modest associations with the risk of CRC in the Czech population (Table 3). The ancestral alleles of two SNPs were associated with an increased risk of CRC: *AGT* rs699 (OR 1.26; 95% CI 1.01-1.57) and *CYP3A7* rs10211 (OR 1.38; 95% CI 1.04-1.83). In contrast, the ancestral allele of *ENPP1* rs1044498 SNP was associated with a decreased risk of CRC (OR 0.79; 95% CI 0.63-1.00). The gene-gene interaction analysis showed no evidence of epistasis (data not shown).

**Table 3:** Genotype distribution of the polymorphisms in the Czech population

**Czech sample population**							
**Gene**	**SNP**	**Allele**	**Genotype distribution (D-DA-A)**		**HWE**	**Dominant model**^**a**^	
**Name**	**ID.**	**(D/A)**	**Controls (N)**	**Cases (N)**	**Controls**	**OR (95% CI)**	**p value**
*AGT*	rs699	T/C	225-354-181	244-492-226	0.07	**1.26 (1.01-1.57)**	**0.04**
*GALNT2*	rs4846914	A/G	291-348-114	372-450-142	0.55	1.03 (0.84-1.27)	0.76
*GALNT2*	rs611229	T/G	314-358-101	426-427-130	0.95	0.89 (0.73-1.09)	0.25
*IFIH1*	rs1990760	T/C	282-355-109	360-455-151	0.87	1.04 (0.85-1.28)	0.72
*SLC2A2*	rs5400	C/T	586-161-14	743-217-18	0.45	1.09 (0.86-1.38)	0.49
*SLC2A2*	rs6785233	T/G	643-120-6	828-146-8	0.88	0.99 (0.76-1.30)	0.95
*SLC2A2*	rs8192675	T/C	392-313-66	491-409-73	0.75	1.03 (0.85-1.26)	0.75
*IGFBP2*	rs1470579	A/C	389-290-82	494-376-104	0.01	1.01 (0.83-1.23)	0.91
*ENPP1*	rs1044498	A/C	573-184-19	773-200-13	0.36	**0.79 (0.63-1.00)**	**0.05**
*ENPP1*	rs9493119	A/G	720-54-1	916-69-5	0.99	1.17 (0.80-1.71)	0.42
*CYP3A[x]*	rs10211	A/G	668-97-4	820-150-8	0.81	**1.38 (1.04-1.83)**	**0.03**
*CYP3A[x]*	rs667660	A/C	668-93-4	830-144-8	0.70	1.24 (0.93-1.65)	0.14
*MTMR9*	rs10091637	T/C	174-394-194	229-496-257	0.34	0.97 (0.77-1.23)	0.79
*MTMR9*	rs11250127	A/G	105-376-290	150-431-388	0.33	0.87 (0.65-1.15)	0.32
*ABCA1*	rs4149268	G/A	309-363-94	398-458-119	0.42	1.02 (0.83-1.25)	0.87
*GAD2*	rs2236418	A/G	529-219-22	667-276-31	0.91	0.93 (0.75-1.15)	0.51
*GAD2*	rs2839670	C/A	526-217-22	672-277-35	0.95	0.94 (0.76-1.16)	0.57
*GAD2*	rs6482538	A/G	648-120-6	812-164-8	0.86	1.03 (0.80-1.35)	0.80
*GAD2*	rs7076544	A/G	520-230-20	645-304-34	0.36	1.02 (0.82-1.25)	0.88
*GAD2*	rs8190748	A/G	400-310-55	501-395-85	0.63	0.98 (0.80-1.19)	0.81
*ERBB3*	rs11171739	T/C	341-301-121	447-354-162	**0.0001**		
*KCTD10*	rs7298565	G/A	158-349-248	185-449-344	0.09	1.19 (0.93-1.53)	0.16
*CYP19A1*	rs12592797	C/A	623-121-7	798-170-9	0.68	1.24 (0.95-1.61)	0.12
*CYP19A1*	rs2445761	A/G	275-352-130	353-456-148	0.34	1.00 (0.82-1.23)	0.98
*CYP19A1*	rs2446405	T/A	527-218-21	672-279-29	0.79	1.11 (0.90-1.38)	0.33
*BBS4*	rs7178130	A/G	280-366-110	388-454-129	0.59	0.88 (0.72-1.08)	0.21
NCP1	rs891286	C/A	145-364-203	194-471-304	0.43	1.09 (0.84-1.40)	0.52

A ancestral allele, D derived allele; N number; HWE Hardy Weinberg equilibrium, OR odds ratio; CI confidence interval; ^a^ adjusted for age and sex; bold numbers indicate statistical significance at 5% level.

**Figure 2:**
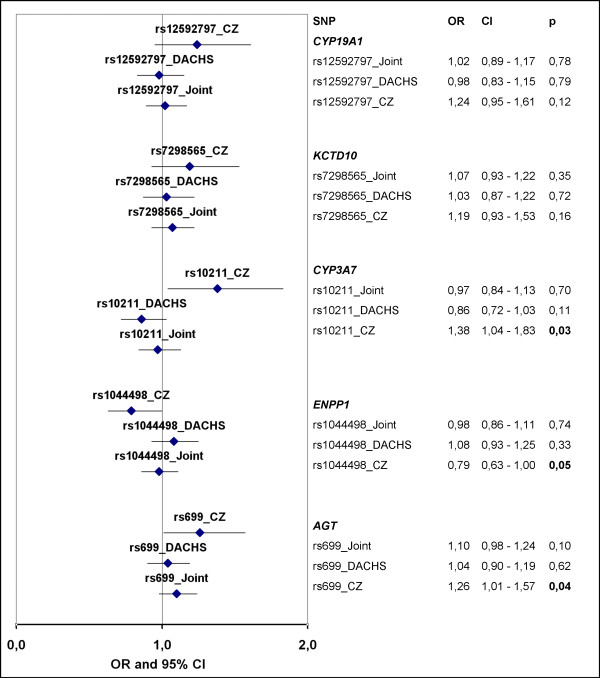
Comparative data plot of the OR and 95% CI of the SNPs analyzed in the Czech and the DACHS cohort; dominant model, individual Czech and DACHS data adjusted for age and sex; joint data adjusted for age and sex, stratified by study; CZ Czech cohort; OR odds ratio; CI confidence interval.

A replication study in the German DACHS population was carried out for the five SNPs with the lowest p values (rs699, rs10211, rs1044498, rs12592797, rs7298565). None of the analysed polymorphisms were associated with the risk of CRC in the German population alone or in the pooled analysis of the two populations (Figure 2).

In the data stratified by sex, *ENPP1* rs1044498 was associated with a decreased risk of CRC in the male subgroup of the Czech population (OR 0.61; 95% CI 0.45 - 0.83; p 0.0015; p_interaction_ 0.01). No association was detected in the Czech female subgroup and in the German study (data not shown).

In the data stratified by BMI, modest associations were detected in the Czech subgroup with a BMI >27 for *AGT* rs699 (OR 1.54; CI 1.05 - 2.25; p 0.027; p_interaction_ 0.036) and *CYP3A7* rs10211 (OR 1.78; CI 1.08 - 2.93; p 0.023; p_interaction_ 0.113). No association was detected in the German study (data not shown).

### Signatures of selection

One important signature of selective pressure was already a criterion to select the SNPs for the study: a high allele frequency difference between HapMap populations. Thus, all SNPs that were associated with the risk of CRC fulfilled this criterion. The highest allele frequency difference among all analysed SNPs was found in *ENPP1* rs1044498 (YRI vs. HCB 94.4%; YRI vs. CEU 87.3%). The SNPs *AGT* rs699 and *CYP3A7* rs10211 showed an allele frequency difference of 52.5% and 67% (YRI vs. CEU), respectively.

**Figure 3:**
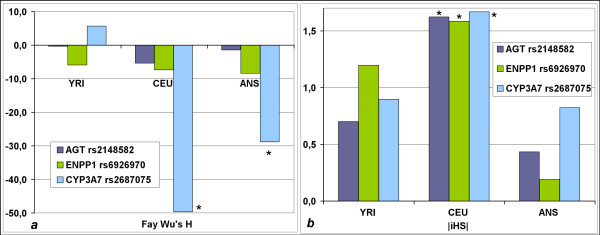
**Plot of Fay-Wu’s H** (***a***) **and plot of the Standardized Integrated Haplotype Score (|iHS|)** (***b***)**. **Estimates for the SNPs that were associated with CRC in the Czech population. Comparison of the African (YRI), European (CEU) and East Asian (ANS) population. All estimates refer to SNPs that are linked to the genotyped SNPs because direct values were not available for the genotyped SNPs. * indicate values that provide conclusive evidence for natural selection [12,15,37].

Fay-Wu’s H and |iHS| were analysed as further signatures of selection. All estimates of Fay-Wu’s H and |iHS| refer to SNPs that are linked to the genotyped SNPs (r^2^=1.0), because direct values were not available in the Haplotter web application http://haplotter.uchicago.edu/[13,15]. Signatures of a selective sweep indicated by Fay-Wu’s H were found for *CYP3A7* rs2687075 (r^2^ to rs10211=1.0), with strong negative H estimates in the ANS population (−28.8) and in the CEU population (−49.6). The other two analysed polymorphisms did not show strong negative H values (> −8.0) or even showed positive values (Figure 3a). Values of |iHS| >1.5 that give conclusive evidence for natural selection in the CEU population were found for SNPs in *AGT* (rs2148582, r^2^ = 1.0 to rs699; |iHS| = 1.62), *ENPP1* (rs6926970, r^2^ = 1.0 to rs1044498; |iHS| = 1.58) and *CYP3A7* (rs2687075, r^2^ = 1.0 to rs10211; |iHS| = 1.67) (Figure 3b).

## Discussion

The present study intended to crosslink susceptibility variants of nutrition-related complex diseases to CRC. In fact, the results in the Czech hospital-based case–control study suggested that polymorphisms in *AGT*, *CYP3A7* and *ENPP1* may be associated with the risk of CRC. However, replication in the population-based German DACHS population did not confirm the associations.

From the 246 SNPs that have been reported to be associated with a nutrition-related disease, 130 showed ancestral susceptibility to overall risk of obesity, T2D, metabolic syndrome or hypertension. However, only 29 SNPs fulfilled the initial selection criterion of ≥45% allele frequency difference between the YRI and any HapMap population, indicating selective pressure. Except *ABCA1* that has been found to be mutated in CRC tumour samples [38,39], none of the 15 genes of the present study has previously been associated with the risk of CRC (http://www.ncbi.nlm.nih.gov/; http://www.hugenavigator.net/ CancerGEMKB/home.do) [40].

The association study in the Czech population indicated ancestral susceptibility to the risk of CRC for the missense *AGT* SNP rs699 and to the 3’UTR SNP rs10211 in *CYP3A7*. Interestingly, SNPs in these two genes feature similar phenotypic effects, such as predisposing to hypertension and salt sensitivity [18].

Published data about *AGT* suggests that the ancestral allele of the probable pathogenic SNP rs699 (M268T), as well as the ancestral alleles of the missense SNP rs4762 (T207M) and the 5′UTR SNP rs5051, predispose to essential hypertension, increased plasma angiotensinogen and increased frequency of preeclampsia (OMIM ID: 189800) [18,41,42]. Additionally, rs5051 (r^2^=0.95 to rs699) has been demonstrated to affect the transcription rate of *AGT*[41,42]. *AGT* (angiotensinogen [serpin peptidase inhibitor, clade A, member 8]) is an important member of the renin-angiotensin system that regulates blood pressure and fluid homeostasis probably through influencing sodium sensitivity [41,42].

Also the intronic *CYP3A5* SNP rs776746 has previously been associated with hypertension and salt sensitivity [9,18]. This SNP has been reported to result in an incorrectly spliced mRNA and in a truncated non-functional protein. In the Czech population, rs776746 was monomorphic. However, the *CYP3A7* SNP rs10211 - also located within the same cytochrome P450 gene cluster and linked to rs776746 in the HapMap CEU population (r^2^=0.82) - showed nominally significant association with the risk of CRC. The genes of the cytochrome P450 gene family encode for some of the most important enzymes involved in the metabolism of various xenobiotics and endogenous substrates such as cholesterol, steroids, environmental carcinogens and drugs [43]. In particular, CYP3 enzymes are responsible for the metabolism of eicosanoids.

The allele frequencies of the genotyped SNPs rs699 and rs10211 and the two functional SNPs rs5051 and rs776746 are highly variable among worldwide populations, with higher frequencies of the derived alleles in non-African populations while the ancestral alleles predominate in the African population. The values of |iHS| determined for SNPs that are fully linked to rs699 and rs10211 provided conclusive evidence for natural selection in the population with European ancestry. Additionally, rs2687075 (r^2^=1.0 to rs10211) in *CYP3A7* showed strong negative values of Fay-Wu’s H that were considered as signatures for a selective sweep in non-African populations [12,15]. Previous studies have already suggested *AGT* and *CYP3A5* as targets of selection, and have additionally connected the two genes directly by their related function [18,42]. In *AGT*, selection was particularly suggested to work on the promoter that contains rs699 and on SNPs in high LD with it. This selection was attributed to altered requirements for the human to maintain sodium homeostasis [42]. Since the derived allele, that predisposes to salt tolerance, is not yet fixed in non-African populations, the remaining ancestral allele, that predisposes to salt sensitivity, shows ancestral susceptibility to related diseases such as hypertension, preeclampsia [41,42] and CRC.

A possible association with the risk of CRC and signatures of recent selection were also observed for one polymorphism in *ENPP1*. However, in contrast to the initial hypothesis, the ancestral allele of rs1044498 was associated with a decreased risk of CRC in the Czech population. *ENPP1* is a member of the ecto-nucleotide pyrophosphatase/phosphodiesterase (ENPP) family. The encoded protein interacts with the insulin receptor thereby inhibiting subsequent signalling. In previous studies, the ancestral allele of the missense polymorphism rs1044498 (Q121K) has been associated with more vivid insulin receptor binding, stronger inhibition of insulin signalling, insulin resistance, an increased risk of T2D and an increased risk of myocardial infarction (OMIM ID: 608446) [9,44,45]. The associations were most pronounced in cohorts that underwent lifestyle interventions to improve an individual’s weight or cholesterol level [45]. It is possible that the effect of *ENPP1* on the risk of metabolic syndrome and the risk of CRC is highly dependent on additional environmental factors or modifiers.

Unfortunately, we were not able to validate our results in the independent German case–control study. Since the Czech and the German populations should not differ significantly in their genetic constitution, differences in nutrition or other environmental factors may contribute to the observed results or the associations may be a chance finding [46,47]. Since the selection of candidate SNPs was based on complex gene-environmental interactions - with the SNP contributing to a phenotype that predisposes to CRC - the detected associations are expected to be weaker than the associations for the original intermediate phenotype. Already in the original reports about the associations of SNPs with nutrition-related diseases, low ORs were detected in most of the cases. As we studied only a few polymorphisms per gene, other polymorphisms with low LD (r^2^<0.8) or rare SNPs (MAF<0.05) that may contribute to the risk of CRC might have been missed. However, considering previously reported associations of several SNPs in the three described genes with components of the metabolic syndrome, the ancestral nature of the risk alleles, and the detected signatures of selection, a true nature of the modest effects on CRC risk in the Czech population cannot be excluded [48]. Especially the close resemblance of the detected associations and function of SNPs in *AGT* and *CYP3A7* may indicate a true effect of the polymorphisms on CRC susceptibility.

## Conclusion

Our study showed evidence of association of the ancestral alleles of polymorphisms in *AGT* and *CYP3A7* and the derived allele of a polymorphism in *ENPP1* with an increased risk of CRC in Czechs, but not in Germans. The ancestral alleles of these SNPs have previously been associated with the nutrition-related diseases hypertension (*AGT* and *CYP3A7*) and insulin resistance (*ENPP1*). Future studies may shed light on the complex genetic and environmental interactions between different types of nutrition-related diseases. The application of additional selection criteria, such as ancestral susceptibility, signatures of selection, or pathway membership, might help to narrow down the numerous published polymorphisms and to find the most promising candidates for association studies. Large study populations that provide the possibility to define large subgroups with specific pre-diagnostic features may be used to review the actual function of such polymorphisms and may provide further insights into the evolution of common complex diseases.

## Competing interests

The authors declare that they have no competing interests.

## Authors’ contributions

The concept and design of the experiments was conceived by SH, AF, and KH. The experiments which provided the basis to this study were performed by SH. Statistical analyses in the association studies and population genetics were performed by SH, MB, AR, RH and AF. The sample collection and database management for the Czech study population was organized and performed by BP, AN, LV, JN and PV. The sample collection and database management for the DACHS study population was organized by MH, HB and JC-C. The manuscript was written by SH and AF. All authors revised the manuscript and contributed to the discussion of the results. The final manuscript was read and approved by all authors.

## Pre-publication history

The pre-publication history for this paper can be accessed here:

http://www.biomedcentral.com/1471-2350/13/94/prepub

## Supplementary Material

Additional file 1 **Shared Ancestral Susceptibility to Colorectal Cancer.pdf. **Title and description of the dataset: Table I. Information about genes and SNPs considered as candidates for the case–control study. Click here for file
