# Supplementary material for: Shared ancestral susceptibility to colorectal cancer and other nutrition related diseases
Source: BMC Med Genet. 2012 Oct 5;13:94. doi: 10.1186/1471-2350-13-94 (PMC3522999; doi:10.1186/1471-2350-13-94)
Supplement: Additional file 1 — Shared Ancestral Susceptibility to Colorectal Cancer.pdf. Title and description of the dataset: Table I. Information about genes and SNPs considered as candidates for the case–control study. [file 1471-2350-13-94-S1.pdf]

Shared Ancestral Susceptibility to Colorectal Cancer and other Nutrition Related Diseases

Authors:

Stefanie Huhn[a,1], Melanie Bevier[a], Anja Rudolph[b], Barbara Pardini[c], Alessio Naccarati[c], Rebecca Hein[b,*], Michael Hoffmeister[d], Ludmila Vodickova[c,e], Jan Novotny[f], Hermann Brenner[d], Jenny Chang-Claude[b], Kari Hemminki[a,g], Pavel Vodicka[c,e], Asta Försti[a,g]

Affiliations:

[a] Department of Molecular Genetic Epidemiology, German Cancer Research Center (DKFZ), Heidelberg, 69120, Germany
[b] Division of Cancer Epidemiology, German Cancer Research Center (DKFZ), Heidelberg, 69120, Germany
[c] Institute of Experimental Medicine, Academy of Sciences of the Czech Republic, Prague 4, 14200, Czech Republic
[d] Division of Clinical Epidemiology and Aging Research, German Cancer Research Center (DKFZ), Heidelberg, 69120, Germany
[e] Institute of Biology and Medical Genetics, 1st Faculty of Medicine, Charles University, Prague 2, 12000, Czech Republic
[f] Department of Oncology, General Teaching Hospital, Prague, 12808, Czech Republic
[g] Center of Primary Health Care Research, Clinical Research Center, Lund University, Malmö, SE-20502, Sweden

[1] Corresponding Author

Stefanie Huhn
German Cancer Research Center (DKFZ); Department of Molecular Genetic Epidemiology
Im Neuenheimer Feld 580; 69120 Heidelberg; Germany
phone: +49 6221 42 1803; fax: +49 6221 42 1810
email: s.huhn@dkfz.de

[*] Current Affiliation of Rebecca Hein: PMV Forschungsgruppe, University of Cologne, Germany

Keywords
Colorectal cancer, nutrition, complex diseases

Introduction to the table

The study focused on SNPs, for which ancestral alleles have previously been associated with nutrition-related complex diseases other than CRC, such as obesity, T2D and metabolic syndrome. Information about such SNPs was collected from 30 published reports by browsing the PubMed database (http://www.ncbi.nlm.nih.gov/sites/entrez?db=pubmed) for the keywords "diabetes", "obesity", "metabolic syndrome" (OMIM ID: 605552) and "hypertension" (OMIM ID: 145500) up to 06/2009. Most of the articles were based on genome-wide association studies or were meta-analyses. A complete list of the publications can be found at the reference list of the additional file1.

Out of these 30 reports, associations with the risk of the diseases and with the related quantitative traits were retrieved. The quantitative traits for diabetes were fasting glucose level and insulin resistance. For obesity, the traits were body mass index (BMI) and waist to hip ratio. The quantitative traits for hypertension and the metabolic syndrome were high-density lipoprotein (HDL) level, low-density lipoprotein (LDL) level, triglycerides level, salt sensitivity, blood pressure and insulin resistance. A complete list of the reported associations can be found the additional file1.

Additional file1: Table I. Information about genes and SNPs considered as candidates for the case-control study.

**Closest Gene (GenBank ID)**	**rs**	**Chr**	**Position**	**SNP (AD)**	**Risk on A or D**	**Associated Disease/Trait [a] (OMIM ID)**	**Function or Location**	**Source**
ABCA1	rs4149274	09q31.01	106.679.485	AG	A	HDL	intron	[1]
ABCA1	rs4149268	09q31.01	106.687.291	TC	A	HDL	intron	[1]
ABCA1	rs1883025	09q31.01	106.704.372	TC	A	HDL	intron	[2]
ABCG8	rs6544713	02p21.00	43.927.635	CT	D	LDL	intron	[2]
ACE	in intron 16	17q23.03		Alu indel	A	HT	intron	[3]
ADAMTS9	rs4607103	03p14.01	64.687.194	CT	A	T2D	ADAMTS9 ~35kb up	[4]
ADD1	rs4961	04p16.03	2.876.755	GT	A	HT	missense	[3]
ADRB2	rs1042714	05q33.01	148.186.916	GC	A	OB	missense	[3]
ADRB3	rs4994	08q12.00	37.943.205	CT	A	BMI, OB, T2D	missense	[3]
AGT	rs699	01q22.02	228.912.667	CT	A	HT	missense	[3, 5]
AGT	rs4762	01q22.02	228.912.850	CT	A	HT	missense	[3, 5]
AGT	rs5051	01q22.02	228.916.745	AG	A	HT	5'UTR	[3, 5]
AHI1	rs1535435	06q23.03	135.798.965	AG	A	T2D	intron	[6]
APOA1-APOC3-APOA 4-APOA5; ZNF259	rs964184	11q23.03	116.154.377	CG	D	HDL,TG	APOA5 ~11kb down; ZNF259 ~100bp down	[1, 2]
APOA1-APOC3-APOA4 -APOA5; ZNF259	rs12286037	11q23.03	116.157.667	CT	D	TG	APOA5 ~9kb down; intron ZNF259	[1]
APOA5/A4/C3/A1	rs486394	11q23.03	116.031.782	AC	D	TG	APOA5 ~134kb down;	[1]
APOA5/A4/C3/A1	rs2000571	11q23.03	116.090.993	GA	D	TG	APOA5 ~74kb down;	[1]
APOA5/A4/C3/A1	rs662799	11q23.03	116.169.167	AG	D	TG	APOA5 ~800bp up	[1]
APOB	rs7557067	02p24.01	21.061.967	AG	A	TG	~16kb up of APOB	[2]
APOB	rs693	02p24.01	21.085.950	CT	D	LDL, CAD	~35kb up	[1, 7]
APOB	rs1713222	02p24.01	21.125.078	CT	A	LDL	~21kb down of APOB	[7]
APOB	rs7575840	02p24.01	21.127.245	GT	D	LDL	~7kb down	[7]
APOB	rs515135	02p24.01	21.139.812	CT	A	LDL	~21kb down of APOB	[1, 2]
APOB	rs562338	02p24.01	21.142.076	CT	D.A.	LDL	~22kb down	[1, 7]
APOB	rs754523	02p24.01	21.165.446	AG	D	LDL, CAD	~45kb down	[1]
APOB	rs4560142	02p24.01	21.237.472	TC	A	LDL	~21kb down of APOB	[7]
APOB	rs4591370	02p24.01	21.237.497	GA	A	LDL	~21kb down of APOB	[7]
APOB	rs538928	02p24.01	21.242.774	AG	D	LDL	~122kb down	[7]
APOB	rs576203	02p24.01	21.247.378	GA	A	LDL	~21kb down of APOB	[7]
APOB	rs488507	02p24.01	21.247.444	GT	D	LDL	~127kb down	[7]
APOB	rs506585	02p24.01	21.250.937	AG	A	LDL	~21kb down of APOB	[7]
APOB	rs478442	02p24.01	21.252.971	TG	A	LDL	~21kb down of APOB	[7]
APOC1	rs4420638	19q13.32	50.115.036	GA	A	LDL, CAD	APOC1 ~590bp up	[1, 2, 7]

APOE	rs429358	19q13.32	50.104.031	CT	A	CAD, LDL	missense	[3]
ATXN2L; SH2B1	rs8049439	16p11.02	28.745.266	CT	A	BMI	ATXN2L intron;<br>SH2B1 ~37kb down	[8]
B3GALT4	rs12695382	06q21.32	120.431.111	AG	D.A.	LDL	intron	[1]
B3GALT4, RXRB,COL11A2	rs2254287	06q21.32	33.252.176	CG	A	LDL	SLC39A7 ~24kb down	[1]
BAZ1B; MLXIPL	rs714052	07q11.23	72.503.055	AG	A	TG	BAZ1B intron;<br>MLXIPL ~142kb down	[2]
BBS4	rs7178130	15q24.01	70.765.505	GA	A	Bardet-Biedl Syndrome (209900), OB	before 5'	[9]
BCAM	rs10402271	19q13.32	50.021.304	GT	A	LDL, CAD	BCAM ~4kb up	[1, 7]
BCAM	rs4605275	19q13.32	50.030.583	CT	A	LDL	BCAM ~13kb up	[7]
BCDIN3D	rs7138803	12q13.13	48.533.985	GA	D	BMI	FAIM2 ~13kb;<br>BCDIN3D ~10kb	[8]
BCL11A	rs10490072	02q16.01	60.523.685	TC	A	T2D	BCL11A ~11kb down	[10]
BCL3	rs4803750	19q13.31	49.939.717	AG	A	LDL	BCL3 ~95kb	[7]
BCL7B; MLXIPL	rs17145738	07q11.23	72.621.060	CT	D.A.	TG	BCL7B ~11kb up;<br>MLXIPL~56kb down	[1]
BDNF; BDNFOS	rs4074134	11p14.01	27,604,111	AG	D	BMI	intron BDNFOS;<br>BDNF~6kb down;	[8]
BDNF; BDNFOS	rs4923461	11p14.01	27,613,736	AG	A	BMI	intron BDNFOS;<br>BDNF~6kb down;	[8]
BDNF; BDNFOS	rs925946	11p14.01	27,624,028	GT	D	BMI	intron BDNFOS;<br>BDNF~6kb down;	[8]
BDNF; BDNFOS	rs10501087	11p14.01	27,626,934	TC	A	BMI	intron BDNFOS;<br>BDNF~6kb down;	[8]
BDNF; BDNFOS	rs6265	11p14.01	27,636,742	GA	A	BMI	missense in BNDF	[8]
C12orf30	rs17696736	12q24.13	110.971.451	AG	D	T2D	Intron	[8]
CACNA1D	rs4687736	03q23.01	53.720.402	CG	A	T2D	intron	[11]
CACNA1D	rs3796347	03q23.01	53.768.180	AG	D	T2D	intron	[11]
CAMK2A	rs3822607	05q33.01	149.592.236	CT	A	T2D	intron	[11]
CAPN10	rs2975760	02q37.03	241.180.086	CT	A	T2D	intron	[4]
CCDC93	rs11684454	02q14.01	118,479,788	AG	D	OB	intron	[4]
CDC123, CAMK1D	rs12779790	10q14.00	12.367.766	AG	D	T2D	CAMK1D ~60kb;<br>CDC123 ~33kb	[4]
CDKAL1	rs10946398	06p23.03	20.769.263	CA	A	T2D	intron	[12, 13]
CDKAL1	rs7754840	06p23.03	20.769.479	GC	D	T2D	intron	[14, 15]
CDKAL1	rs9465871	06p23.03	20.825.484	TC	D	T2D	intron	[10]
CDKN2B	rs564398	09p21.03	22.019.797	TC	A	T2D	intron	[11]
CDKN2B	rs10811661	09p21.03	22.124.344	CT	D	T2D	CDKN2BAS ~12kb up	[12-16]
CELSR2	rs6657811	01p13.03	109.609.056	AT	A	LDL	intron	[7]
CELSR2	rs4970834	01p13.03	109.616.653	CT	A	LDL	intron	[7]
CELSR2	rs611917	01p13.03	109.617.025	CT	D	LDL	intron	[7]

CELSR2	rs12740374	01p13.03	109.619.363	GT	A	LDL	3'UTR	[2, 7]
CELSR2	rs660240	01p13.03	109.619.611	AG	D	LDL	3'UTR	[7]
CELSR2	rs629301	01p13.03	109.620.079	AC	A	LDL	down 3' CELSR2	[7]
CELSR2	rs646776	01p13.03	109.620.303	AG	A	LDL	down 3' CELSR2	[7]
CELSR2	rs602633	01p13.03	109.623.284	AC	D	LDL	4kb down CELSR2; up of 5' PSRC1	[1, 7]
CELSR2	rs599839	01p13.03	109.623.939	GA	D	LDL	down 3' CELSR2; up of 5' PSRC1	[1, 7]
CETP	rs9989419	16q13.00	55.542.890	GA	D	HDL	CETP~10kb down	[1]
CETP	rs173539	16q13.00	55.545.795	TC	D	HDL	CETP ~8kb down	[2]
CETP	rs3764261	16q13.00	55.551.075	CA	A	HDL	CETP ~3kb down	[1]
CETP	rs1864163	16q13.00	55.554.984	GA	D	HDL	intron	[1]
CHGA	rs941584	14q32.12	92.461.325	AG	A	T2D	intron	[11]
Chr 02 gap	rs6712932	02q12.01	105.204.280	AG	D	T2D	GPR45 ~20kb down	[17]
Chr 11 gap	rs9300039	11p12.00	41.872.192	CA	A	T2D	API5 ~1Mb down	[15]
Chr 12q13	rs11171739	11q13.02	54.757.142	CT	A	T2D	ERBB3 ~4kb down	[10]
Chr 1p13	rs6679677	01p13.02	114.105.581	CA	D	T2D	-	[10]
Chr 21 gap	rs200801	21q21.01	18.255.767	AG	A	T2D	mRNA's ~80kb up; Chondrolectin ~300kb down	[6]
Chr 21 gap	rs2254434	21q21.01	18.257.252	GA	D	T2D	mRNA's ~80kb up; Chondrolectin ~300kb down	[6]
Chr 21 gap	rs158081	21q21.01	18.291.122	AG	D	T2D	gene gap	[6]
CILP2	rs16996148	09p13.11	19.519.722	GT	A	LDL, TG	CLIP2 ~1kb up	[1]
CILP2,PBX4	rs17216525	09p13.11	19.523.470	CT	A	TG	CILP2 ~5kb up; PBX5 ~67kb down	[2]
CLEC16A	rs12708716	16p13.13	11.087.624	AG	D	T2D	intron	[10]
CLEC2D	rs3764021	12p13.31	9.725.145	GA	D	T2D	cds synon	[10]
CTLA4	rs3087243	02q33.02	204.447.414	GA	D	T2D	down <1kb	[3]
CYP19A1 / GLDN	rs2446405	15q21.02	49.434.335	AT	A	IR	GLDN intron	[18]
CYP3a5	rs776746	07q22.01	99.108.725	AG	A	HT; salt sensitivity	intron	[3, 5]
DCD	rs1153188	12q13.02	53.385.513	AT	A	T2D	DCD ~60kb up	[4]
DOCK7	rs1748195	01p31.03	62.822.431	GC	D	TG	intron	[1]
DOCK7	rs10889353	01p31.03	62.891.034	AC	A	TG	intron	[2]
DPEP2	rs255052	16q22.01	66,582,746	GA	A	HDL	intron	[1]
EGFR	rs2075112	07p11.02	55.187.355	GA	D	T2D	intron	[11]
ENPP1	rs1044498	06q23.02	132.214.311	CA	A	IR	missense	[3]
ETV5; DGKG	rs7647305	03q27.02	187.317.234	TC	D	BMI	ETV5 ~8kb up; DGKG ~30kb down;	[8]
EXT2	rs1113132	11p11.02	44.210.229	CG	A	T2D	intron	[16]
EXT2	rs11037909	11p11.02	44.212.440	CT	D	T2D	intron	[16]

EXT2	rs3740878	11p11.02	44.214.628	AG	A	T2D	intron	[16]
FADS1-FADS2-FADS3	rs174547	11q12.02	61.327.609	CT	A	HDL,TG	intron	[2]
FGF2; NUDT6	rs1048201	04q28.01	124.034.008	CT	D	T2D	3'UTR in FGF2; missense in NUDT6	[11]
FOXA3	rs11669442	19q13.32	51.066.647	TC	D	T2D	intron	[11]
FTO	rs6499640	16q12.02	52.327.428	AG	A	BMI	intron	[8]
FTO	rs9939973	16q12.02	52.358.319	GA	D	OB	intron	[19]
FTO	rs1421085	16q12.02	52.358.705	TC	D	BMI	intron	[20]
FTO	rs1121980	16q12.02	52.366.998	TC	A	BMI, OB, T2D	intron	[19, 21]
FTO	rs8050136	16q12.02	52.374.026	AC	A	BMI	intron	[8]
FTO	rs8050136	16q12.02	52.374.026	AC	A	T2D, BMI	intron	[12, 13, 15]
FTO	rs3751812	16q12.02	52.376.211	GT	D	BMI	intron	[8]
FTO	rs9939609	16q12.02	52.377.778	AT	A	BMI	intron	[22]
FTO	rs9939609	16q12.02	52.378.278	AT	A	T2D, BMI	intron	[10]
FTO	rs7190492	16q12.02	52.386.503	GA	A	BMI	intron	[8]
FTO	rs9930506	16q12.02	52.388.216	GA	A	BMI, WtH	intron	[23]
FTO	rs8044769	16q12.02	52.396.886	CT	A	BMI	intron	[8]
G6PC2	rs560887	02q24.03	169.471.644	GA	D	FGL	intron	[10]
GAD2	rs2236418	10p12.01	26.545.752	GA	A	OB	5'UTR	[3]
GALNT2	rs2144300	01q42.13	228.361.789	CT	A	HDL, TG	intron	[1]
GALNT2	rs4846914	01q42.13	228.362.564	GA	A	HDL	intron	[1, 2]
GCA	rs3788964	02q24.02	162.915.976	AG	D	T2D	intron	[24]
GCK	rs4607517	07p13.00	44.202.443	GA	D	FGL	GCK ~7kb up;	[24]
GCKR	rs1260326	02p23.03	27.584.694	CT	D	TG	missense	[1, 2]
GCKR	rs780094	02p23.03	27.594.991	CT	D	TG	intron	[1]
GNPDA2	rs10938397	04p13.00	44.877.534	AG	D	BMI, OB, T2D	GNPDA2 ~454kb up	[21]
GRIN3A	rs1323432	09q31.01	103.403.008	AG	D	HDL	intron	[1]
HHEX	rs1111875	10q23.33	94.453.112	TC	D	T2D	HHEX ~8kb up	[13-16]
HHEX	rs5015480	10q23.33	94.455.789	CT	A	T2D	HHEX ~10kb up	[12, 13]
HHEX	rs7923837	10q23.33	94.472.147	GA	A	T2D	HHEX ~27kb up	[16]
HLA-DQA1	rs9272346	06p21.32	32.712.600	AG	D	T2D	HLA-DQA1 ~500bp down	[10]
HLA-DRB1	rs9270986	06p21.32	32.682.288	CA	D	T2D	HLA-DRB1 ~28kb up	[10]
HMGCR	rs3846663	05q13.03	74.691.732	TC	A	LDL	intron	[2]
HNF1A	rs2650000	12q24.31	119.873.595	CA	D	LDL	HNF1A ~28kb down	[2]
HNF1B	rs757210	17q12.00	33.170.878	AG	A	T2D	intron	[12]
HNF1B	rs7501939	17q12.00	33.175.519	CT	D	T2D	intron	[11]
HNF4A	rs1800961	20q13.12	42.476.028	CT	D	HDL	missense	[2]
IFIH1	rs1990760	02q24.02	162.832.547	CT	A	T2D	missense	[10]

IGF2BP2	rs4402960	03q27.02	186.994.631	GT	D	T2D	intron	[12-16]
IGF2BP2	rs1470579	03q27.02	187.012.024	CA	A	T2D	intron	[14]
IL2RA	rs706778	10p15.01	6.139.205	AG	A	T2D	intron	[10]
IL2RA	rs2104286	10p15.01	6.139.301	AG	D		intron	[10]
INS	rs689	11p15.05	2.139.050	TA	A	T2D	intron	[10]
INSIG2	rs7566605	02q14.01	118.552.745	GC	D	OB	INSIG2 ~10kb down	[17]
JAZF1	rs864745	07p15.01	28.147.331	TC	A	T2D	intron	[4]
KCNJ11	rs5219	11p15.01	17.366.398	TA (ACGT)	A	T2D	nonsense	[10, 12, 14, 15]
KCNJ12	rs5215	11p15.01	17.365.456	TC	D		missense	[10, 13]
KCTD10; MYO1H	rs2338104	12q24.11	108.379.801	GC	A	HDL	KCTD10 intron; MYO1H ~9kb up	[1, 2]
KCTD15	rs29941	19q13.11	39.001.622	CT	A	BMI	KCTD15 ~6kb up	[8]
KCTD15	rs11084753	19q13.11	39.014.227	GA	A	BMI, OB, T2D	KCTD15 ~17kb up	[21]
KIAA1109	rs6534347	04q27.00	123.418.135	GA	D	T2D	intron	[10]
LDLR	rs11668477	19p13.02	11.056.280	AG	A	LDL	LDLR ~4kb down	[7]
LDLR	rs6511720	19p13.02	11.063.556	GT	A	LDL; CAD	intron	[1, 2]
LDLR	rs2228671	19p13.02	11.072.162	TC	D	LDL	cds synon	[7]
LIPC	rs4775041	15q22.01	56.462.237	GC	A;D	TG; HDL	LIPC ~45kb down	[1]
LIPC	rs10468017	15q22.01	56.466.054	CT	A;D	HDL	LIPC ~45kb down	[1, 2]
LIPC	rs261332	15q22.01	56.514.867	AG	D.A.	HDL	intron	[1]
LIPG	rs4939883	18q21.01	45.421.462	TC	A	HDL	LIPIG ~49kb up	[1, 2]
LIPG	rs2156552	18q21.01	45.435.916	AT	D	HDL	LIPIG ~63kb up	[1]
LOC387761	rs7480010	11p12.00	42.203.544	GA	A	T2D	API5 ~1Mb down	[16]
LOC441171	rs9494266	06q23.03	135.893.516	AG	A	T2D	AHI1 up ~33kb	[6]
LPL	rs2197089	08p21.03	19.870.903	GA	D.A.	HDL, TG	LPL ~2kb up	[1]
LPL	rs12678919	08p21.03	19.888.752	AG	D.A.	HDL,TG	LPL ~18kb up	[1, 2]
LPL	rs10503669	08p21.03	19.892.220	CA	A	HDL, TG	LPL ~23kb up	[1]
LPL	rs6993414	08p21.03	19.947.448	AG	A	TG	LPL ~79kb up	[1]
LPL	rs6586891	08p21.03	19.959.128	AC	D	HDL, TG	LPL ~90kb up	[1]
MAF	rs1424233	16q23.02	78.240.502	AG	A	BMI	MAF ~48kb up	[20]
MAFB	rs6102059	20q12.00	38.662.448	CT	A	LDL	MAFB ~86kb down	[2]
MC4R	rs17782313	18q21.32	56.002.327	TC	D	BMI	MC4R ~187kb down	[20]
MC4R	rs12970134	18q21.32	56.035.980	GA	D	BMI, T2D	MC4R ~53kb down	[8]
MTCH2	rs4752856	11p11.02	47.604.868	GA	D	BMI, OB, T2D	intron	[21]
MTCH2	rs10838738	11p11.02	47.619.875	AG	D	BMI	intron	[1]
MTMR9	rs2293855	08p23.01	11.215.070	GA	A	OB	intron	[25]
MTNR1B	rs1387153	11q21.00	92.313.726	CT	D	FGL	MTNR1B ~29kb down	[26]
MTNR1B	rs10830963	11q21.00	92.348.608	CG	D	FGL, Early insulin response to glucose	intron	[24, 27]

MYO10	rs31313	05p15.01	16.744.004	AG	A	T2D	intron	[11]
MYO10	rs173738	05p15.01	16.779.130	GA	D	T2D	intron	[11]
MYO10	rs253336	05p15.01	16.893.891	CA	D	T2D	intron	[11]
NCAN	rs2228603	09p13.11	19.191.174	CT	A	LDL	missense	[1]
NEGR1	rs3101336	01p31.01	72.524.023	AG	D	BMI	NERG1 ~3kb up	[8]
NEGR1	rs2568958	01p31.01	72.537.954	GA	D	BMI	NERG1 ~17kb up	[8]
NEGR1	rs2815752	01p31.01	72.585.278	CT	D	BMI, OB, T2D	NERG1 ~64kb up	[21]
NFATC1	rs643705	19q23.00	75.262.909	CG	?	T2D	intron	[11]
NFATC1	rs3826567	19q23.00	75.300.716	CT	D	T2D	intron	[11]
NFKB1	rs230498	04q24.00	103.708.889	GA	A	T2D	intron	[11]
NFKB1	rs230539	04q24.00	103.714.820	AG	A	T2D	intron	[11]
NFKB1	rs11722146	04q24.00	103.743.917	AG	A	T2D	intron	[11]
NFKB1	rs1609798	04q24.00	103.756.738	CT	A	T2D	intron	[11]
NLRC5	rs1566439	16q13.00	55.582.413	TC	A	HDL	intron	[11]
NOTCH2	rs10923931	01p12.00	120.319.732	GT	D	T2D	intron	[11]
NPC1	rs1805081	18q11.02	19.394.680	AG	A	BMI	missense	[20]
NRXN3	rs10146997	14q31.01	79.015.165	GA	A	BMI, WtH	intron	[28]
NUTF2	rs2271293	16q22.01	66.459.821	GA	A	HDL	intron	[2]
PAX6	rs628224	11p13.00	31.776.000	GA	A	T2D	intron	[11]
PBX1	rs7535186	01q23.03	162.834.520	GA	D	T2D	intron	[11]
PCSK1	rs6235	05q15.00	95.754.904	GC	D	BMI, OB, T2D	missense	[3, 9, 29]
PCSK1	rs6232	05q15.00	95.777.791	AG	D	BMI, OB, T2D	missense	[9, 29]
PCSK9	rs11206510	01p32.03	55.268.877	CT	D	LDL, CAD	PCSK9 ~9kb down	[1, 2]
PFPK	rs6602024	10p15.02	3.145.487	GA	D	BMI	intron	[23]
PLTP	rs7679	20q13.12	44.010.159	TC	D	HDL,TG	3' UTR	[2]
PPARG	rs1801282	03p25.02	12.368.375	CG	A	T2D	intron / nonsense	[11]
PRL	rs4712652	06p22.03	22.186.844	AG	A	BMI	PRL ~208kb up	[20]
PSRC1	rs599839	01p13.03	109.623.939	GA	D	LDL, CAD	up of 5' PSRC1	[1, 7]
PTER	rs10508503	10p13.00	16.340.207	CT	A	BMI	PTER ~179kb down	[20]
PTPN22	rs2476601	01p13.02	114.179.341	GA	D	T2D	missense	[10]
PTPN23	rs6679677	01p13.02	114.105.581	CA	D	T2D	RSBN1~45kb down	[10]
RAB11B	rs2967605	19p13.02	8.375.988	CT	D	HDL	RAB11B ~17kb up	[2]
RBKS	rs11127129	02p23.02	27.930.462	CG	A	TG	intron	[1]
SEC16B	rs10913469	01q25.02	176.180.392	CT	A	BMI	intron	[8]
SF4; NCAN; TM6SF2	rs10401969	09p13.11	19.268.968	TC	A	TG	SF4 intron;<br>NCAN ~45kb up;<br>TM6F2 ~24kb up	[1, 2]
SH2B1	rs4788102	16p11.02	28.781.149	GA	D	BMI	SH2B1 ~1,4kb down	[8]

SH2B1	rs7498665	16p11.02	28.790.992	GA	A	BMI, OB, T2D	missense	[21]
SLC12A3	rs12596776	16q13.00	55.477.099	CG	A	HDL	intron	[1]
SLC2A2	rs5400	03q26.02	172.215.244	TC	A	T2D	missense	[3]
SLC30A8	rs13266634	08q24.11	118.254.214	CT	A	T2D	missense	[12-16]
STK32C	rs7910485	10q26.03	133.899.573	GA	D	T2D	intron	[6]
SYN2, PPARG	rs17036101	03p25.02	12.253.095	AG	D	T2D	SYN2 ~45kb up;<br>PPARG ~51kb down	[3, 10, 12, 14, 15]
TCF7L2	rs17747324	10q25.02	114.742.743	TC	D	T2D	intron	[15]
TCF7L2	rs7901695	10q25.02	114.744.328	GA	A	T2D	intron LD to 7903146	[6, 10, 12, 15]
TCF7L2	rs4506565	10q25.02	114.746.281	TA	A	T2D	intron	[10]
TCF7L2	rs7903146	10q25.02	114.748.589	TC	A	T2D	intron	[10, 14-16, 30]
TCF7L2	rs12255372	10q25.02	114.799.142	CA	D	T2D	intron	[6, 30]
THADA	rs7578597	02p21.00	43.586.577	TC	A	T2D	missense	[4]
TIMD4-HAVCR1	rs1501908	05q33.03	156.330.997	CG	D	LDL	TIMD4 ~8kb up	[2]
TMEM18	rs2867125	02p24.03	613.077	GA	A	BMI	TMEM18 ~45kb down	[8]
TMEM18	rs6548238	02p24.03	625.155	CT	D	BMI, OB, T2D	TMEM18 ~33kb down	[21]
TMEM18	rs4854344	02p24.03	628.394	GT	D	BMI	TMEM18 ~30kb down	[8]
TMEM18	rs7561317	02p24.03	635.203	AG	D	BMI	TMEM18 ~23kb down	[6]
TOMM40	rs2075650	19q13.31	50.087.709	GA	A	LDL	intron	[7]
TRIB1	rs17321515	08q24.13	126.555.841	AG	A	TG	TRIB1 ~36kb upsream	[1]
TRIB1	rs2954029	08q24.13	126.560.404	AT	A	TG	TRIB1 ~41kb upsream	[1, 2]
TSPAN8	rs7961581	12q21.01	69.949.619	TC	D	T2D	TSPAN8 ~112kb up	[4]
TTC39B	rs471364	09p22.03	15.279.828	TC	D	HDL	intron	[2]
TTC7B	rs942740	14q32.01	90.228.872	GA	D	T2D	intron	[6]
TTC7B	rs1749718	14q32.01	90.253.330	AG	A	T2D	intron	[6]
USF1	rs3737787	01q23.03	159.276.397	CT	A	Familial combined hyperlipidemia (144250)	3'UTR	[3]
VEGFA	rs9472138	06p21.01	43.919.990	CT	D	T2D	VEGFA ~58kb up	[4]
WFS1	rs752854	04p16.01	6.333.112	AG	A	T2D	intron	[11]
WFS1	rs10010131	04p16.01	6.344.066	GA	D	T2D	intron	[12]
WFS1	rs10010131	04p16.01	6.344.066	GA	A	T2D	intron	[11]
WFS1	rs6446482	04p16.01	6.346.844	CG	?	T2D	intron	[11]
WFS1	rs734312	04p16.01	6.354.505	GA	D	T2D	missense	[11]
XKR6-AMAC1L2	rs7819412	08p23.01	11.082.821	AG	A	TG	intron	[2]

[a] Next to the keywords that were used to collect and select the candidate SNPs from the literature, the table also includes all reported associations with related quantitative traits.

Chr chromosome; A ancestral allele; D derived allele; BMI body mass index (OMIM ID: 606641); T2D type 2 diabetes (OMIM ID: 125853); OB obesity (OMIM ID: 601665); HT hypertension (OMIM ID: 145500); TG triglycerides; LDL low-density lipoprotein; HDL high-density lipoprotein; CAD coronary atery disease (OMIM ID:

608320); IR insulin resistance ; FGL fasting glucose level ; WtH waist to hip ratio ; UP upstream ; DOWN downstream; Chromosomal band according to UCSC Genome Browser (Feb. 2009 (GRCh37/hg19) Assembly) [31-33], Chromosomal position according to ref_assembly at ncbi dbSNP (http://www.ncbi.nlm.nih.gov).

References

1. Willer CJ, Sanna S, Jackson AU, Scuteri A, Bonnycastle LL, Clarke R, Heath SC, Timpson NJ, Najjar SS, Stringham HM *et al*: **Newly identified loci that influence lipid concentrations and risk of coronary artery disease**. *Nat Genet* 2008, **40**(2):161-169.
2. Kathiresan S, Willer CJ, Peloso GM, Demissie S, Musunuru K, Schadt EE, Kaplan L, Bennett D, Li Y, Tanaka T *et al*: **Common variants at 30 loci contribute to polygenic dyslipidemia**. *Nat Genet* 2009, **41**(1):56-65.
3. Di Rienzo A, Hudson RR: **An evolutionary framework for common diseases: the ancestral-susceptibility model**. *Trends Genet* 2005, **21**(11):596-601.
4. Zeggini E, Scott LJ, Saxena R, Voight BF, Marchini JL, Hu T, de Bakker PI, Abecasis GR, Almgren P, Andersen G *et al*: **Meta-analysis of genome-wide association data and large-scale replication identifies additional susceptibility loci for type 2 diabetes**. *Nat Genet* 2008, **40**(5):638-645.
5. Thompson EE, Kuttab-Boulos H, Witonsky D, Yang L, Roe BA, Di Rienzo A: **CYP3A variation and the evolution of salt-sensitivity variants**. *Am J Hum Genet* 2004, **75**(6):1059-1069.
6. Salonen JT, Uimari P, Aalto JM, Pirskanen M, Kaikkonen J, Todorova B, Hypponen J, Korhonen VP, Asikainen J, Devine C *et al*: **Type 2 diabetes whole-genome association study in four populations: the DiaGen consortium**. *Am J Hum Genet* 2007, **81**(2):338-345.
7. Sandhu MS, Waterworth DM, Debenham SL, Wheeler E, Papadakis K, Zhao JH, Song K, Yuan X, Johnson T, Ashford S *et al*: **LDL-cholesterol concentrations: a genome-wide association study**. *Lancet* 2008, **371**(9611):483-491.
8. Thorleifsson G, Walters GB, Gudbjartsson DF, Steinthorsdottir V, Sulem P, Helgadottir A, Styrkarsdottir U, Gretarsdottir S, Thorlacius S, Jonsdottir I *et al*: **Genome-wide association yields new sequence variants at seven loci that associate with measures of obesity**. *Nat Genet* 2009, **41**(1):18-24.
9. Benzinou M, Walley A, Lobbens S, Charles MA, Jouret B, Fumeron F, Balkau B, Meyre D, Froguel P: **Bardet-Biedl syndrome gene variants are associated with both childhood and adult common obesity in French Caucasians**. *Diabetes* 2006, **55**(10):2876-2882.
10. The.Wellcome.Trust CCC: **Genome-wide association study of 14,000 cases of seven common diseases and 3,000 shared controls**. *Nature* 2007, **447**(7145):661-678.
11. Sandhu MS, Weedon MN, Fawcett KA, Wasson J, Debenham SL, Daly A, Lango H, Frayling TM, Neumann RJ, Sherva R *et al*: **Common variants in WFS1 confer risk of type 2 diabetes**. *Nat Genet* 2007, **39**(8):951-953.
12. McCarthy MI, Zeggini E: **Genome-wide association scans for Type 2 diabetes: new insights into biology and therapy**. *Trends Pharmacol Sci* 2007, **28**(12):598-601.
13. Zeggini E, Weedon MN, Lindgren CM, Frayling TM, Elliott KS, Lango H, Timpson NJ, Perry JR, Rayner NW, Freathy RM *et al*: **Replication of genome-wide association signals in UK samples reveals risk loci for type 2 diabetes**. *Science* 2007, **316**(5829):1336-1341.
14. Saxena R, Voight BF, Lyssenko V, Burtt NP, de Bakker PI, Chen H, Roix JJ, Kathiresan S, Hirschhorn JN, Daly MJ *et al*: **Genome-wide association analysis identifies loci for type 2 diabetes and triglyceride levels**. *Science* 2007, **316**(5829):1331-1336.
15. Scott LJ, Mohlke KL, Bonnycastle LL, Willer CJ, Li Y, Duren WL, Erdos MR, Stringham HM, Chines PS, Jackson AU *et al*: **A genome-wide association study of type 2 diabetes in Finns detects multiple susceptibility variants**. *Science* 2007, **316**(5829):1341-1345.
16. Sladek R, Rocheleau G, Rung J, Dina C, Shen L, Serre D, Boutin P, Vincent D, Belisle A, Hadjadj S *et al*: **A genome-wide association study identifies novel risk loci for type 2 diabetes**. *Nature* 2007, **445**(7130):881-885.
17. Herbert A, Gerry NP, McQueen MB, Heid IM, Pfeufer A, Illig T, Wichmann HE, Meitinger T, Hunter D, Hu FB *et al*: **A common genetic variant is associated with adult and childhood obesity**. *Science* 2006, **312**(5771):279-283.
18. Lo JC, Zhao X, Scuteri A, Brockwell S, Sowers MR: **The association of genetic polymorphisms in sex hormone biosynthesis and action with insulin sensitivity and diabetes mellitus in women at midlife**. *Am J Med* 2006, **119**(9 Suppl 1):S69-78.
19. Hinney A, Nguyen TT, Scherag A, Friedel S, Bronner G, Muller TD, Grallert H, Illig T, Wichmann HE, Rief W *et al*: **Genome wide association (GWA) study for early onset extreme obesity supports the role of fat mass and obesity associated gene (FTO) variants**. *PLoS One* 2007, **2**(12):e1361.

20. Meyre D, Delplanque J, Chevre JC, Lecoeur C, Lobbens S, Gallina S, Durand E, Vatin V, Degraeve F, Proenca C *et al*: **Genome-wide association study for early-onset and morbid adult obesity identifies three new risk loci in European populations**. *Nat Genet* 2009, **41**(2):157-159.
21. Renström F, Payne F, Nordstrom A, Brito EC, Rolandsson O, Hallmans G, Barroso I, Nordstrom P, Franks PW: **Replication and extension of genome-wide association study results for obesity in 4923 adults from northern Sweden**. *Hum Mol Genet* 2009, **18**(8):1489-1496.
22. Frayling TM, Timpson NJ, Weedon MN, Zeggini E, Freathy RM, Lindgren CM, Perry JR, Elliott KS, Lango H, Rayner NW *et al*: **A common variant in the FTO gene is associated with body mass index and predisposes to childhood and adult obesity**. *Science* 2007, **316**(5826):889-894.
23. Scuteri A, Sanna S, Chen WM, Uda M, Albai G, Strait J, Najjar S, Nagaraja R, Orru M, Usala G *et al*: **Genome-wide association scan shows genetic variants in the FTO gene are associated with obesity-related traits**. *PLoS Genet* 2007, **3**(7):e115.
24. Prokopenko I, Langenberg C, Florez JC, Saxena R, Soranzo N, Thorleifsson G, Loos RJ, Manning AK, Jackson AU, Aulchenko Y *et al*: **Variants in MTNR1B influence fasting glucose levels**. *Nat Genet* 2009, **41**(1):77-81.
25. Yanagiya T, Tanabe A, Iida A, Saito S, Sekine A, Takahashi A, Tsunoda T, Kamohara S, Nakata Y, Kotani K *et al*: **Association of single-nucleotide polymorphisms in MTMR9 gene with obesity**. *Hum Mol Genet* 2007, **16**(24):3017-3026.
26. Bouatia-Naji N, Bonnefond A, Cavalcanti-Proenca C, Sparso T, Holmkvist J, Marchand M, Delplanque J, Lobbens S, Rocheleau G, Durand E *et al*: **A variant near MTNR1B is associated with increased fasting plasma glucose levels and type 2 diabetes risk**. *Nat Genet* 2009, **41**(1):89-94.
27. Lyssenko V, Nagorny CL, Erdos MR, Wierup N, Jonsson A, Spegel P, Bugliani M, Saxena R, Fex M, Pulizzi N *et al*: **Common variant in MTNR1B associated with increased risk of type 2 diabetes and impaired early insulin secretion**. *Nat Genet* 2009, **41**(1):82-88.
28. Heard-Costa NL, Zillikens MC, Monda KL, Johansson A, Harris TB, Fu M, Haritunians T, Feitosa MF, Aspelund T, Eiriksdottir G *et al*: **NRXN3 is a novel locus for waist circumference: a genome-wide association study from the CHARGE Consortium**. *PLoS Genet* 2009, **5**(6):e1000539.
29. Kilpelainen TO, Bingham SA, Khaw KT, Wareham NJ, Loos RJ: **Association of variants in the PCSK1 gene with obesity in the EPIC-Norfolk study**. *Hum Mol Genet* 2009, **18**(18):3496-3501.
30. Scott LJ, Bonnycastle LL, Willer CJ, Sprau AG, Jackson AU, Narisu N, Duren WL, Chines PS, Stringham HM, Erdos MR *et al*: **Association of transcription factor 7-like 2 (TCF7L2) variants with type 2 diabetes in a Finnish sample**. *Diabetes* 2006, **55**(9):2649-2653.
31. King DC, Taylor J, Elnitski L, Chiaromonte F, Miller W, Hardison RC: **Evaluation of regulatory potential and conservation scores for detecting cis-regulatory modules in aligned mammalian genome sequences**. *Genome Res* 2005, **15**(8):1051-1060.
32. Kolbe D, Taylor J, Elnitski L, Eswara P, Li J, Miller W, Hardison R, Chiaromonte F: **Regulatory potential scores from genome-wide three-way alignments of human, mouse, and rat**. *Genome Res* 2004, **14**(4):700-707.
33. Kong A, Gudbjartsson DF, Sainz J, Jonsdottir GM, Gudjonsson SA, Richardsson B, Sigurdardottir S, Barnard J, Hallbeck B, Masson G *et al*: **A high-resolution recombination map of the human genome**. *Nat Genet* 2002, **31**(3):241-247.
